# Gel-free genotyping of deletion alleles in *Caenorhabditis elegans* with real-time PCR

**DOI:** 10.17912/micropub.biology.000274

**Published:** 2020-07-11

**Authors:** Keon Wimberly, Keith P Choe

**Affiliations:** 1 Genetics Institute, University of Florida, Gainesville, FL, USA; 2 Department of Biology, University of Florida, Gainesville, FL, USA

**Figure 1:**
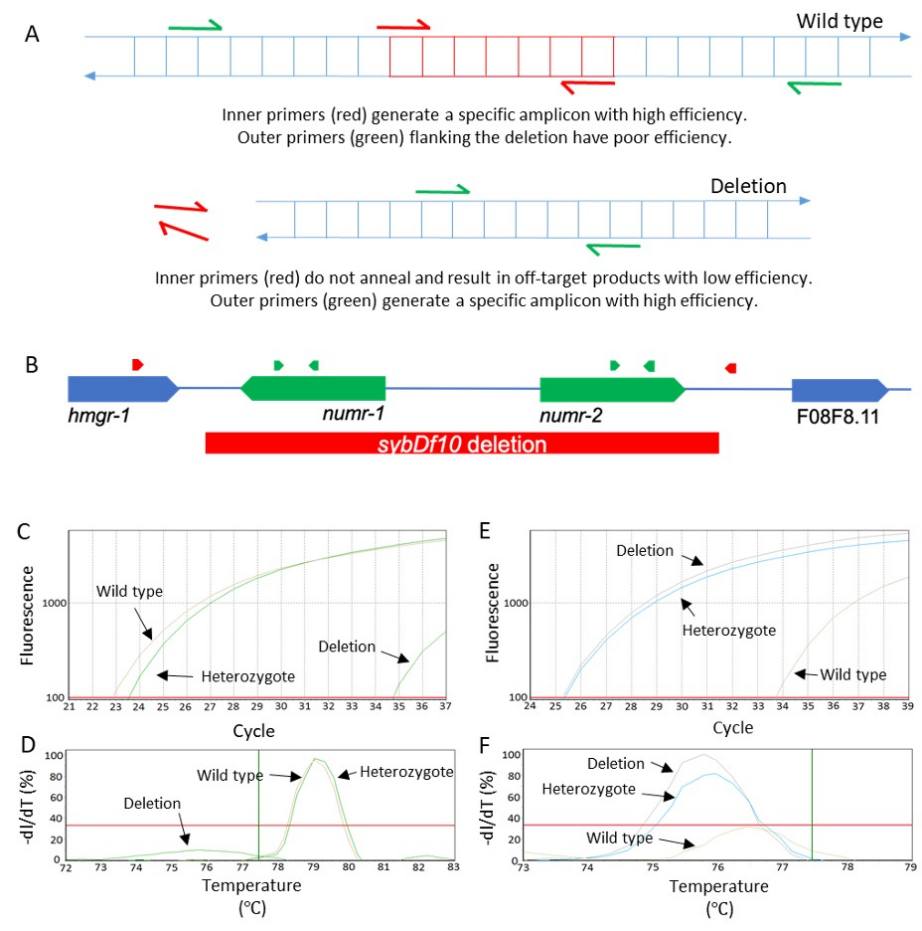
A) Schematic of primer design approach; the deletion is red. B) Genomic map of the *numr-1 & -2* deletion. C-F) Real-time PCR amplification and melt curves for inner primers (C-D) and outer (E-F) primers.

## Description

*C. elegans* is a powerful model system for studying the genetic basis of biological processes. Identifying relevant genes and organizing them into genetic pathways often involve analyzing phenotypes when mutations for two or more genes are combined in the same strain. This requires crossing and genotyping resulting individual F2 lineages. In the simplest case, mutant alleles cause robust phenotypes that can be observed with a microscope. Alleles without easily observable phenotypes can be followed by including closely linked alleles with obvious phenotypes (Wang and Sherwood 2011; Fay 2013). In some cases, neither of these scenarios are possible, which leaves the mutated allele as the only factor for selection.

The traditional method of genotyping deletion alleles in *C. elegans* involves PCR amplification and detection of distinct product sizes after electrophoresis (Liu *et al.* 1999; Ahringer 2006). This method is reliable and permits visualization of product lengths, but requires additional steps to cast, load, run and visualize gels after PCR. Laboratories that study gene expression commonly use real-time PCR systems for quantitative RT-PCR. Here, we tested if real-time PCR could also be used to screen worm DNA lysates for deletion alleles. Real-time PCR systems detect accumulation of products in each well during cycling using double-stranded specific DNA dyes and arrays of illuminators and photodetectors; amplification of product can be viewed during cycling. Cycle threshold (Ct) is calculated automatically by real-time PCR systems as the earliest cycle that fluorescence exceeds background and is inversely related to log of starting template concentration. After the last PCR cycle, fluorescence is monitored in each well over a range of temperatures to produce a melting curve that can help distinguish different amplicons.

In theory, real-time PCR should be able to detect the 2:1 ratio in template DNA concentration between homozygous and heterozygous worms. However, a quantitative approach to identify heterozygotes would require biological replication and amplification of a separate normalizing gene to be reliable (Radonić *et al.* 2004; Zhang *et al.* 2012). We instead devised a qualitative approach where wild type and deletion alleles are detected for each sample in separate DNA-dye reactions run together on the same plate in a real-time PCR machine. Wild type ‘inner’ primers amplify a portion of the sequence within the deletion and mutant ‘outer’ primers amplify sequence flanking the deletion (Figure 1A).

By running these two parallel reactions in the same real-time PCR plate, we are able to distinguish wild type, heterozygous, and homozygous mutant genotypes at the end of a single real-time PCR run. Over the last year, we have used this approach for five deletions alleles. As proof of concept, we present data using a *numr-1 & -2(sybDf10)* deletion allele (Figure 1B-F). It is also worth mentioning that a portion of the intergenic region downstream from *numr-2* and upstream from F08F8.11 is also deleted, which explains the ‘df’ designation. NUclear localized Metal Responsive-1 *(numr-1 & -2)* is a gene duplicate pair that is involved in RNA metabolism and is highly induced by heavy metals (Tvermoes, Boyd, and Freedman 2010; Wu *et al.* 2019). N2 was used as a wild type control, and a simulated heterozygous sample was generated by mixing equal volumes of *numr-1 & -2* and N2 lysates.

Inner primers for a 142 bp amplicon resulted in Ct values less than 30 (Figure 1C) and similar melting curves (Figure 1D) with wild type and heterozygous lysates. These same inner primers resulted in a Ct value over 10 cycles greater and a sub-threshold melting curve with homozygous mutant lysates; these results likely represent primer-dimer or off-target products. Outer primers flanking the deletion for a 102 bp amplicon (in the mutant background) resulted in Ct values less than 30 (Figure 1E) and similar melting temperatures (Figure 1F) with homozygous mutant and heterozygous lysates; these same outer primers resulted in Ct values over 8 cycles greater and a sub-threshold melting curve with wild type lysates. These results demonstrate that homozygous wild type, homozygous deletion, and heterozygous genotypes are easily distinguished by comparing Ct values.

Our approach is robust and saves time by avoiding electrophoresis, but there are important limitations. Real-time PCR is best suited to following previously characterized deletions after crossing; it does not provide information on product size, and therefore electrophoresis is better suited to characterizing novel deletions. Costs are decreasing, but real-time PCR systems typically cost substantially more than traditional PCR and gel electrophoresis systems. Therefore, the approach we present is best suited to laboratories that already have a real-time PCR system for measuring gene expression. If a machine is already available, then the added costs of a DNA dye during PCR and a second reaction for each lysate are offset by avoiding the costs of gels and gel stains. After lysis and mixing of reactions, final results are available at the end of hands-free real-time PCR and melt curve programs. Both traditional and real-time PCR reagents and machines are now available that can complete reactions in less than hour with optimization (Nayab *et al.* 2008).

## Methods

**Design two pairs of primers (Figure 1).** Primers can be designed using Primer-BLAST (https://www.ncbi.nlm.nih.gov/tools/primer-blast/), which can screen against off-target annealing in the *C. elegans* genome. Inner primers should anneal within the deletion. Outer primers should flank the deletion. Primers can be designed to either coding or non-coding regions, but coding region primers amplified more consistently in our hands. We typically design amplicons to be 75-250 bps for their targeted genotype. Multiple primer pairs that satisfy these criteria can be prepared, validated, and used to confirm genotypes. Prepare primers according to standard real-time PCR protocols.

**Worm preparation.** Isolate individual hermaphrodite L4 stage worms into separate agar plates or wells and allow them to molt and lay at least 50 eggs. If she can be found easily, the single gravid mother can be picked for genotyping and the remaining offspring from the same plate can be recovered for downstream experiments. Alternatively, five or more offspring from each mother can be picked as a sample of the brood. It is possible to unknowingly pick only homozygous deletion worms from a heterozygous mother, but this is chance is small (0.25^5^ = 1/1024), decreases exponentially by picking additional worms, and genotypes can be verified in a candidate lineage by testing a larger sample of the population.

**Prepare worm lysates.** Lysis buffer is 10 mM Tris pH 8, 1.0% Triton X-100 detergent, 1% Tween 20, and 0.5 mM EDTA (Ly *et al.* 2015). Store lysis buffer without proteinase K in aliquots at -20°C. On the day of worm picking, add proteinase K to a final concentration of 2 mg/mL. For each sample, add 5 μL of water to the lid of a 0.2 mL PCR tube; note that it is more difficult to pick worms to the bottom of tubes. Place worms into the water; a mild detergent like Triton X-100 can be added at 0.2% to reduce sticking of worms to plastic (Peter R. Boag, personal communication). After worms are added to lids, add 5 μL of complete lysis solution to the bottom of the same PCR tubes. Close the lids and centrifuge in a mini centrifuge (1,000 to 2,000 x g) for 30 seconds to collect worms in the bottom. Observe the tubes under a microscope to ensure that worms are at the bottom. Freeze samples at -80°C for at least 10 minutes.

**Lyse worms.** Remove sample tubes from the freezer and immediately place them in a thermal cycler programmed at 65°C for 90 minutes to activate proteinase K. After 60 minutes, pull some of the tubes out of the thermal cycler, check for complete lysis with a microscope, and return them. There should be no visible worms remaining; if there are, there may be too many worms, or you may need to remake lysis buffer or use fresh proteinase K. After lysis, incubate the samples at 95°C for 10 minutes to inactivate proteinase K. Dilute the lysates with DNase-free water to dilute detergents, worm debris, and other biomolecules that could interfere with real-time PCR; with our system and master mix, 1/5 or 1/10 dilutions work well.

**Real-time PCR.** Use a real-time PCR master mix and follow optimized and recommended procedures to prepare a sufficient volume for your reactions. We use Forget-Me-Not™ qPCR Master Mix by Biotium (Cat # 31041-T), but master mixes can also be made from recipes to save costs (Karsai *et al.* 2002). Add 8 μL of qPCR master mix (with primers) and 2 μL of diluted lysate to each well and seal the plate with optically clear tape. Place the plate in a real-time thermal cycler. Program the first step at 95°C for 2 minutes to activate DNA polymerase. Add a two-step cycle that is repeated 40 times: 95°C for 5 seconds (denaturization) and 60°C for 25 seconds (annealing and elongation). The annealing and elongation step can be extended proportionately according to amplicon length. Add a melting curve analysis as the final step.

**Analysis.** Validating new primers requires wild type, simulated heterozygote, and (known) homozygous mutant lysates as controls. Mix equal volumes of wild type and mutant lysates to generate a simulated heterozygote. Lysates from homozygote wild type mothers or offspring populations should only yield a robust and specific product (Ct value less than 30 and prominent melt curve) with inner primers. Lysates from homozygote deletion mothers or offspring populations should only a yield a specific product with outer primers. Lysates from heterozygote mothers or offspring populations should yield a specific product with both primer pairs. If double mutants are not viable as homozygotes, then they will not be identified among the F2 lineages despite ample sampling. After these criteria are satisfied, primers can be used to screen genotypes of F2 generations after crossing. Rerun control lysates on the same plate as unknown samples to verify that primers and master mixes are working properly.
